# HLA-DQ2/8 and COVID-19 in Celiac Disease: Boon or Bane

**DOI:** 10.3390/microorganisms11122977

**Published:** 2023-12-13

**Authors:** Aaron Lerner, Carina Benzvi, Aristo Vojdani

**Affiliations:** 1 The Zabludowicz Center for Autoimmune Diseases, Chaim Sheba Medical Center, Ramat Gan 5262160, Israel; carina.ben.zvi@gmail.com; 2 Research Department, Ariel University, Ariel 4077625, Israel; 3 Immunosciences Lab., Inc., Los Angeles, CA 90035, USA; drari@msn.com

**Keywords:** celiac disease, human leukocyte antigen, HLA-I, HLA-II, SARS-CoV-2, COVID-19, protective genes, gastrointestinal tract

## Abstract

The SARS-CoV-2 pandemic continues to pose a global threat. While its virulence has subsided, it has persisted due to the continual emergence of new mutations. Although many high-risk conditions related to COVID-19 have been identified, the understanding of protective factors remains limited. Intriguingly, epidemiological evidence suggests a low incidence of COVID-19-infected CD patients. The present study explores whether their genetic background, namely, the associated HLA-DQs, offers protection against severe COVID-19 outcomes. We hypothesize that the HLA-DQ2/8 alleles may shield CD patients from SARS-CoV-2 and its subsequent effects, possibly due to memory CD4 T cells primed by previous exposure to human-associated common cold coronaviruses (CCC) and higher affinity to those allele’s groove. In this context, we examined potential cross-reactivity between SARS-CoV-2 epitopes and human-associated CCC and assessed the binding affinity (BA) of these epitopes to HLA-DQ2/8. Using computational methods, we analyzed sequence similarity between SARS-CoV-2 and four distinct CCC. Of 924 unique immunodominant 15-mer epitopes with at least 67% identity, 37 exhibited significant BA to HLA-DQ2/8, suggesting a protective effect. We present various mechanisms that might explain the protective role of HLA-DQ2/8 in COVID-19-afflicted CD patients. If substantiated, these insights could enhance our understanding of the gene–environment enigma and viral–host relationship, guiding potential therapeutic innovations against the ongoing SARS-CoV-2 pandemic.

## 1. Introduction

The COVID-19 pandemic has been a global health concern for more than the last three years. Its worldwide geographical propagation, contagious ability, multi-faceted clinical presentation, prognosis, morbidity, mortality, and short/long-term outcome has driven the scientific and clinical teams to study the disease’s geoepidemiology, in terms of susceptible environmental [1,2] and genetic predisposing factors [3,4]. In contrast to predisposing factors, several immune-biological parameters (decreased ACE2 expression, increased anti-inflammatory cytokines, antibody response, and T cell activation), environmental factors (healthy diet, sufficient nutrition, atopic conditions, and COVID-19 vaccination) or genetic polymorphisms, were reported to attenuate COVID-19 severity [5,6].

One of the most explored human genetic factors related to the SARS-CoV-2 infection is the human leukocyte antigen (HLA) genotype [7,8,9]. In humans, the HLA comprises a complex of genes on the 6th chromosome that coordinate and regulate the immune systems by encoding cell-surface proteins. Being a major and indispensable vital antigen-presenting pathway, they play a pivotal role in establishing susceptibility to infectious conditions. Their crucial role in adaptive immunity impacts and their vast diversity in the human genome dictates the quality and strength of the reactive immune response to hostile pathogens [10]. The HLAs’ distinct sequences and three-dimensional structures influence peptide binding efficiency and determine the adaptive immune response. In fact, facing a viral infection, the HLA allelic repertoire impacts the transmission, phenotype, symptomatic intensity and outcome of the invading virus. The same holds for the actual COVID-19 viruses [11] and specific vaccine development against SARS-CoV-2 [12].

Most probably, individual susceptibility to the SARS-CoV-2 virus and its corresponding COVID-19 disease is determined by the host HLA genes’ variations [7,8]. The wide range of the infected hosts and the innate and reactive immune responses to SARS-CoV-2 is HLA allele-dependent. No less important are the relationships between HLA polymorphisms and multiform COVID-19 disease presentations, courses, and outcomes. The cross-talks between the HLAs’ vast repertoire and the virus are more complicated since SARS-CoV-2 was recently established as an auto-immunogenic virus [13,14,15,16,17], and autoimmune diseases are heavily HLA allele-dependent [18]. Interestingly, most recently, the predisposition to autoimmune diseases was expanded to the post-COVID vaccine syndromes [17,19], thus augmenting the concerns regarding a future increase in the incidence of those conditions [20].

Multiple reports exist on the relations between HLA variants and COVID-19 susceptibility and outcome [3,4,7,8]. Based on some new observations, the present study concentrates on the potential protective effects of HLA polymorphisms on the SARS-CoV-2 infection. Furthermore, the dual role played by the specific HLA-DQ2 and 8 in celiac disease (CD) susceptibility is discussed. HLA-DQ2 and 8 are the major genetic predisposing factors for CD [21,22]. In fact, the preferential binding of HLA-DQ2 (90–95%) and HLA-DQ8 (5–10%) to gluten peptides provides a strong inherited basis for CD. On the other hand, the same haplotypes were recently suggested to protect CD patients from the COVID-19 disease [23]. It seems that those HLAs represent a double-edged sword in the CD–COVID-19 interplay.

The aims of the current study were: 1. To summarize the literature on CD–HLA–SARS-CoV-2 interrelations, highlighting the CD-protective HLA genes, 2. To explore sequence alignment between SARS-CoV-2 epitopes, CCC antigens and HLA-DQ2/8, and 3. To suggest potential mechanisms for HLA-DQ2/8 protection from COVID-19.

### 1.1. Protective HLA Alleles in Celiac Disease

The topics of protective HLA polymorphisms related to autoimmune diseases in general [24,25] and, more specifically, to SARS-CoV-2 infectivity have been extensively reported [8,26,27,28,29,30,31,32,33,34,35,36], despite some controversary on the latter topic [37]. Interestingly, some HLA variants were described to confer resistance to CD development. Logically, a severe, morbid, and lethal autoimmune disease, like CD, before gluten was discovered as the offending environmental inducer, should have decreased in incidence or even disappeared along the course of human evolution [38,39]. Intriguingly, its incidence is consistently increasing, mounting to 1–2% in many countries [40,41]. Table 1 summarizes the CD-protective HLA variants.

It can be concluded that multiple HLAs are protective toward CD. 

### 1.2. The Human Gastrointestinal Tract Is a Target Organ for SARS-CoV-2

Despite the plethora of extraintestinal manifestations of CD [52,53,54], its primary target organ is the small bowel [55,56]. Indeed, SARS-CoV-2 preferentially infects the upper respiratory tract, but the gastrointestinal tract (GIT) and its associated organs are no less affected [57,58,59,60,61,62,63,64]. Table 2 summarizes the enteric aspects that were described during the COVID-19 pandemic.

It is concluded that the SARS-CoV-2 virus penetrates, infects, overcomes the gut protective barrier mechanisms, damages the enteric mucosa, activates the mucosal immune systems, is excreted in the feces, is transmitted feco–orally, and is spread into the surrounding environment. It should be stressed that those events were not explored in connection to the CD–SARS-CoV-2 interplay.

### 1.3. COVID-19-Celiac Disease Interplay

The COVID-19–CD relationship is characterized by a typically wobbly movement, considering risks and morbidity. The pendulum went from potential risk [58], no increased risk at all for COVID-19 [61,82,83,84,85,86,87,88,89], lower incidence of CD diagnosis during the present pandemic compared to previous years [90], to increased impact of the COVID-19 lockdown and restrictions on CD therapy [91,92] and potential life-threatening delay in CD diagnosis [55,93]. The elderly CD patients are susceptible to a more complicated and stormier course of COVID-19 due to their gut functional senescence and associated comorbidity [61,89]. Most recently, an increase in new-onset childhood CD during the COVID-19 pandemic was described [94]. In contrast, the risk of hospitalization is mitigated by anti-COVID-19 vaccinations [95]. Intriguingly, a potential outbreak of CD during the COVID-19 epidemic or in the post-COVID era, in the forthcoming future, was lately hypothesized [96,97]. However, this pessimistic outcome was most recently contradicted by Greco N et al. [23].

Based on the above, it seems that the topic of the risk of COVID-19 in CD patients is highly controversial. Until a systemic review and a meta-analysis will be performed, the jury is not out yet. Since it was suggested that the CD-associated HLA DQ2/DQ8 haplotype might have a protective role against COVID-19 infection [23], the aims of the present study are to explore this relationship by analyzing sequence similarities between known SARS-CoV-2 epitopes and CCC, and identifying epitopes with significant BA to HLA-DQ2 and DQ8. 

Our hypothesis is that increased sequence similarity to CCC and specific CD-associated HLA BA to the virus epitopes elicit a greater protective production of anti-SARS-CoV-2 antibodies, resulting in full protection or attenuated disease course. 

## 2. Materials and Methods

### 2.1. Data Sources

For this study, our primary data were obtained from two distinct databases, CCC Protein Sequences (protein sequences related to CCC were extracted from the UniProt Knowledgebase [98], accessible via https://www.uniprot.org/ on 17 August 2023), and SARS-CoV-2 Epitope Data (extracted from the Immune Epitope Database and Analysis Resource (IEDB) as of 17 August 2023 [99], accessible via https://www.iedb.org/ on 18 August 2023). 

#### 2.1.1. Protein Sequences Extraction of Common Cold Coronaviruses

Protein sequences corresponding to various CCC strains were obtained from the UniProt Knowledgebase, including OC43 (Taxon ID 31631), HKU1 (Taxon ID 443239 Isolate N1), NL63 (Taxon ID 277944), and 229E (Taxon ID 11137). Of particular interest, due to their recognized immunodominance in clinical research, were the spike glycoprotein, nucleoprotein, and certain non-structural proteins (NSPs) from replicase polyprotein 1ab—specifically, NSP3, NSP4, NSP12, and NSP13 [100,101]. They correspond to the following UniProt IDs: Q5MQD0, P36334, Q6Q1S2, P15423, Q5MQC6, P33469, Q6Q1R8, P15130, P0C6X2, P0C6X5, P0C6X6, and P0C6X1.

#### 2.1.2. Data Retrieval from IEDB

IEDB data were retrieved on 17 August 2023, using the specific filters mentioned below:

- Organism: SARS-CoV-2 (ID: 2697049).
- Antigens: Spike glycoprotein (P0DTC2), nucleoprotein (P0DTC9), replicase polyprotein 1ab (P0DTD1).
- Epitope structure: Linear sequence.
- Assay type: Only positive assays pertaining to T-cell epitopes and MHC ligands were considered. This refers to epitopes validated through laboratory experiments.
- MHC restriction: Class II.
- Host: Human.
- Diseases: No specific restrictions; all diseases were considered.

Around six thousand SARS-CoV-2 epitope entries were extracted from the IEDB, focusing on MHC-II ligands and T-cell assays.

### 2.2. Sequence Similarity Identification

Using the EMBOSS Matcher, a robust Pairwise Local Alignment tool [102,103], sequence similarities between SARS-CoV-2 epitopes and CCC protein sequences were identified. Based on Bill Pearson’s Lalign application algorithm (version 2.0u4, February 1996), the Matcher reveals local sequence resemblances. As a cutoff, we only considered sequences that displayed at least 11 identical amino acids (AAs) within a 15-mer epitope span. This threshold draws upon findings by Mateus et al., where an epitope homology exceeding 67% between SARS-CoV-2 and human CCC resulted in CD4 T cells’ cross-reactivity in 57% of the instances [104]. This analysis pinpointed 924 unique immunodominant 15-mer epitopes from SARS-CoV-2 with a minimum of 67% similarity to at least one human CCC strain under examination.

### 2.3. Binding Affinity Prediction to HLA-DQ2, DQ8

**Figure 1:**
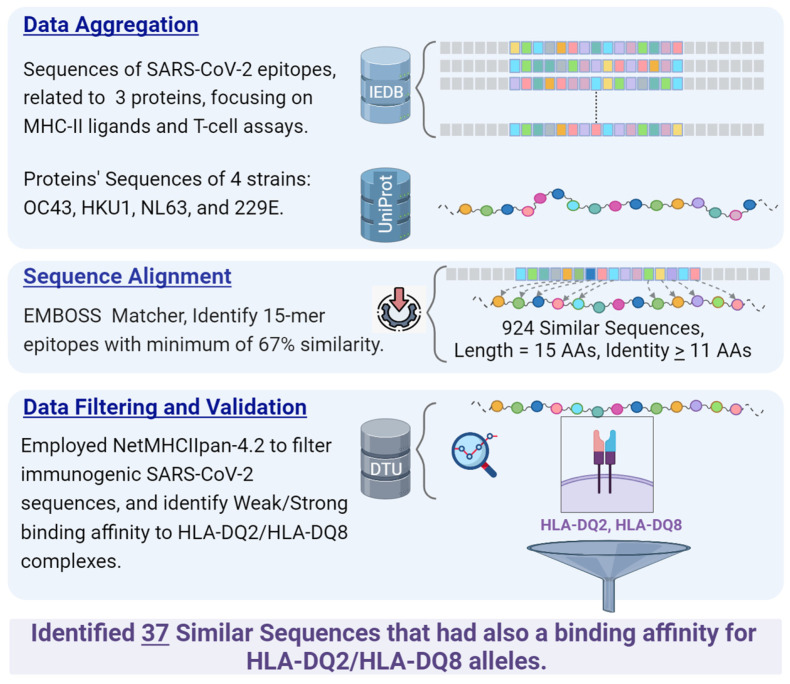
A graphical representation of the workflow for searching sequence similarity and HLA-DQ2/HLA-DQ8 binding affinity. Data Aggregation: SARS-CoV-2 epitopes were extracted from IEDB. UniProt was searched to retrieve proteins sequences of four CCC strains, OC43, HKU1, NL63, and 229E. Sequence Alignment: Emboss Matcher was employed; 924 similar sequences were found with a cut-off ≥11 identical AAs on 15-mer sequences. Data Validation: NetMHCIIpan-4.2 method was employed on the 924 sequences, and 37 were found to have a significant BA to HLA-DQ2/DQ8. Created with BioRender (accessible via https://www.BioRender.com/ on 5 November 2023).

Given that immunodominant SARS-CoV-2 epitopes are associated with the capacity to bind to multiple HLA allelic variants, we aimed to scrutinize the 924 epitopes’ BA to HLA-DQ2 and HLA-DQ8 complexes. These complexes are encoded by the leukocyte histocompatibility antigen genes DQA1*05:01–DQB1*02:01 and DQA1*03:01–DQB1*03:02, respectively, both located on chromosome 6p21, and are key factors in predisposing individuals to CD [105]. We employed predictive tools from DTU Health Tech, specifically, the NetMHCIIpan-4.2 method, which is accessible at DTU Health Tech Services. The 4.2 version offers superior predictive accuracy and broader molecular coverage, particularly as an HLA-DQ data model [106]. The model provides a %Rank score, which predicts how likely a peptide will naturally bind to a selected HLA receptor. This score is normalized based on predictions from a random peptide set and it indicates where their predicted BA stands in relation to a distribution derived from these random natural peptides. Specifically, we use a %Rank of less than 5% as our cutoff to determine significant binding, either “strong” or “weak” binders. Epitopes with a %Rank below 1% are categorized as strong binders (SB), while those with rankings between 1% and 5% are termed weak binders (WB). The flowchart in Figure 1 presents a comprehensive overview of our methodology.

## 3. Results

A total of 6301 SARS-CoV-2 epitope entries were sourced from IEDB. Many of these epitopes were identified using multiple assays: 3249 through T-cell assays and 11,851 as MHC-II ligand assays. Out of these, 924 distinct 15-mer SARS-CoV-2 epitopes were identified, displaying at least 67% similarity with the listed human CCC strains [104]. These epitopes were predominantly found in the immunologically prevalent proteins: spike glycoprotein, nucleoprotein, and specific immunodominant non-structural proteins (NSPs) from replicase polyprotein 1ab, such as NSP3, NSP4, NSP12, and NSP13 [100].

**Figure 2:**
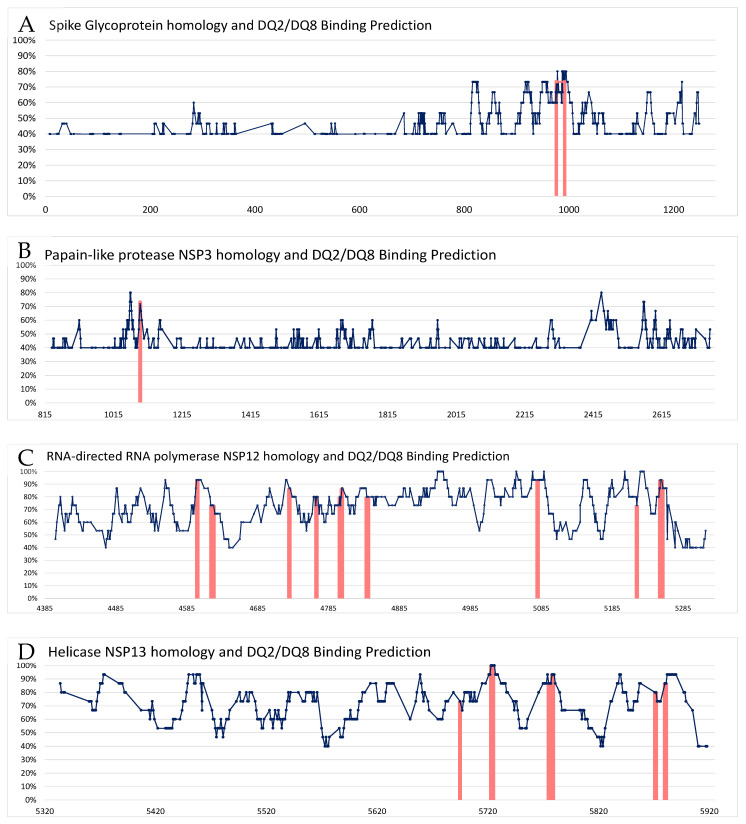
Homology between SARS-CoV-2 and CCC epitope sequences, in relation to HLA-DQ2/8. Each panel represents a specific protein: (**A**) Spike glycoprotein, (**B**) NSP3, (**C**) NSP12, and (**D**) NSP13. The dark blue lines indicate the percentage of homology between SARS-CoV-2 and the highest matching CCC (among 229E, NL63, HKU1, and OC43). Only similarities of 40% or above are shown. Regions displaying significant binding to HLA-DQ2/HLA-DQ8 alleles are highlighted in red.

Upon applying a computational BA prediction tool (NetMHCIIpan-4.2) to the 924 epitopes, 37 showed considerable BA to either HLA-DQ2 or HLA-DQ8 alleles. Delving deeper, 3 were associated with the spike Glycoprotein (all WB), 1 with NSP3 (WB), 20 with NSP12 (3 SB, 17 WB), and 13 with NSP13 (1 SB, 12 WB). Neither the nucleoprotein nor NSP4 exhibited epitopes with notable BA to HLA-DQ2 or HLA-DQ8 alleles. Importantly, one epitope, DKVEAEVQIDRLITG, echoed findings from Obermair et al. and correlated positively with HLA-DQA103:01/DQB103:02 alleles [107]. Given the multiple studies suggesting pre-existing immune memory to SARS-CoV-2 antigens in unexposed individuals [104,108], the present results might indicate that the presence of HLA-DQ2/HLA-DQ8 alleles could facilitate the activation and proliferation of CD4 T cells that cross-react with SARS-CoV-2 epitopes. In Figure 2, the homology levels of each SARS-CoV-2 15-mer epitope are compared with CCC epitope sequences, highlighting those with a similarity of 40% or higher. Each figure corresponds to a specific protein, with regions with high similarity (above 67%) and significant BA to HLA-DQ2/HLA-DQ8 alleles marked in red. 

Table 3 illustrates the 15-mer SARS-CoV-2 epitopes that not only display at least 67% homology with CCCs but also have a significant BA for HLA-DQ2/HLA-DQ8 alleles. The two columns on the right-hand-side display the binding rank. Those ranked in the top 1% were classified as potential SB and those ranked in the top 5% were classified as WB.

## 4. Discussion

By screening the IEDB, 924 distinct 15-mer SARS-CoV-2 epitopes were identified, displaying at least 67% similarity with the listed human CCC strains. Applying the computational BA prediction tool, 37 of those epitopes exposed a considerable BA to either HLA-DQ2 or HLA-DQ8 alleles (20 associated with NSP12, 13 with NSP13, 3 with the spike glycoprotein, and 1 with NSP3). Higher sequence similarity and a more effective affinity to specific HLAs were associated with a higher antibody response, thus conferring more efficient protection against a virus [8,23,26,27,28,29,30,31,32,33,34,35,36,104,108]. A similar explanation can explain the fact that CD patients are relatively protected against COVID-19. The present results add a new dimension to the CD–COVID-19 cross-talks. The HLA-DQ2/8 can represent a double-edged sword, being the most prevalent CD-susceptible genes, hence protective ones against SARS-CoV-2. 

It is well accepted that more than 95% of celiacs have the HLA-DQ2 (DQA1*05:01–DQB1*02:01, abbreviated as DR3-DQ2) and a minority carries the DQ8 haplotype (DQA1*03:01–DQB1*03:02, abbreviated as DR4-DQ8) [21]. In fact, those genetic markers can help in substantiating the diagnosis of CD in uncertain cases, considering its negative predictive value [109]. They can help in following the genetic family tree of affected members [110]. Additionally, they may assist in predicting or discriminating individuals at high risk of CD like first- and second-degree relatives, or associated conditions such as various autoimmune diseases or specific genetic disorders (Down, Turner, or Williams syndromes) [111].

In contrast to those predisposing CD-associated HLA-DQs, a new hypothesis was most recently forwarded by Greco N, et al. [23], suggesting a protective role against COVID-19 in CD-affected populations. The authors found that only 5.8% of their 191 active and non-active CD-tested population were positive for SARS-CoV-2, with most of them exhibiting no or mild symptoms and never hospitalized.

The protective role of HLAs against bacteria [112], parasites [113], and even viruses is well reported. Their role in fighting viruses like HIV [114], HCV [115], HBV [116], and SARS [8,26,27,28,29,30,31,32,33,34,35,36,117,118,119] have been documented. Interestingly, the protective role of the CD-associated HLA II suggested by Greco N et al. [23] represents a refreshing new idea that can help in our understanding of the celiac–COVID-19–HLA axis. The low incidence of the COVID-19-infected CD patients and their a/hypo-symptomatic presentation have been discussed in numerous publications on the celiac’s resilience to SARS-CoV-2 infection [61,82,83,84,85,86,87,88]. Interestingly, several other non-celiac-associated HLAs are associated with asymptomatic (HLA-B*15:01, HLA-DRB1*04:01) or milder forms (HLA-B*15, HLA-DRB1*04) of COVID-19 [120,121].

Several mechanisms can be speculated to explain this HLA-DQ2 and 8, COVID-19 protection in CD patients:

1. High affinity between SARS-CoV-2 antigens and HLA-DQ immune presentation to the T cells enhances anti-SARS-CoV-2 immunity. In fact, the HLA allele most associated with COVID-19 deterioration is HLA-A*11. However, HLA II also plays a role in the disease severity, with HLA-DRB1*15:01 and HLA-DRB1*04 alleles being examples [122]. Unfortunately, the CD-associated HLA-DQs were not explored when the binding affinities of 438 HLA alleles were screened [122]. Nevertheless, asymptomatic and mildly/moderately affected patients likely develop an effective early immune response to clear the virus [123]. A reasonable explanation for the associations between CD, SARS-CoV-2, and HLA-DQ2/8 observed presently is that most of the strong HLA binders of coronavirus peptides are also strong binders of other sequences, and hence, are likely to be general strong binders that probably underwent selection in the past [122].
2. SARS-CoV-2-naïve people might have a certain measure of HLA-dependent immune defense presented by antibodies cross-reactive to other CCC [124]. Most of those HLAs belong to HLA class I, hence, a minority of them are part of class II. Unfortunately, the CD-associated HLA-DQ were not explored [124]. The topic of a potential protective cross-reactivity against the COVID-19 virus in uninfected CD patients conferred by their HLA-DQ2/8 is a subject for further investigation.
3. An individual HLA variant has its unique repertoire of peptides with a specific sequence structure to stick in the peptide-binding groove of HLA. It appears that certain HLA haplotypes have higher preferences to present peptides with specific molecular functions [125]. This HLA preferential presentation was extrapolated to explain the protective effect of certain HLA alleles in infectious diseases, including COVID-19. Indeed, Karnaukhov V. et al. reported on HLA-A/HLA-B and HLA-A/HLA-C variants having a more distinct functional antigen preference presentation, but the HLA-DQ2/8 ones were not explored [125]. The authors reported on HLA differential presentation of SARS-CoV-2 antigens mainly by HLA type I alleles, hence, the CD-associated HLA-DQ haplotypes might play a protective or attenuative role in COVID-19 disease. Notably, several studies reported on HLA-DQ variants associated with a dominant T cell response against the SARS-CoV-2 virus, resulting in a milder disease [120,126,127], including a higher production of antibodies post mRNA-based vaccination [128].
4. Cross-reactive antibodies shared between SARS-CoV-2 and gluten. If cross-reactivity exists between the virus and gluten, those reactive antibodies might attenuate the severity of COVID-19 and protect the untreated or the non-compliant CD patients. In fact, Vojdani A, et al. reported on such cross-reactive antibodies [129]. Screening 180 different food antigens and peptides, the authors showed that SARS-CoV-2 proteins share cross-reactive epitopes with various food antigens that had not been previously explored. Wheat and alpha-gliadin were shown to cross-react with SARS-CoV-2 spike protein and nucleoprotein [129]. More so, the authors reported on sequence similarity between SARS-CoV-2 proteins and alpha-gliadin toxic peptides and glutenin, thus, reinforcing a potential effect of the COVID-19–food axis relationships. It should be stressed that the potential protective effects of the above-mentioned cross-reactive antibodies and the sequence similarity were not substantiated and should be further evaluated.
5. Increase in anti-inflammatory factors in COVID-19-infected celiac patients. Recently, Asri N et al. studied naïve CD patients for various inflammatory and anti-inflammatory markers [85]. The CD patients exposed an increased expression of anti-inflammatory molecules like CD4, CD25 (IL-2Rα), and FOXP3, compared to severe COVID-19 patients and controls. However, the HLA-DQs’ allelic status was not investigated. The increase in the anti-inflammatory profile might be beneficial to the CD patients by lowering COVID-19 severity and attenuating the disease course. The relationship of those markers to the HLA-DQs should farther be explored.
6. HLA-DQ2/8 might be important in fighting human viruses. The mechanism of CD risk modification by HLA heterogeneity might involve differential presentation of autoantigenic sequences by HLA class II proteins. The HLA-DQ2 and DQ8 presentation of viral epitopes were reported concerning coxsackievirus-specific peptides [130]. The authors speculated that the phenomenon might represent a protective adaptive mechanism to maximize anti-enterovirus responses. The same can be speculated for the COVID-19 virus and the HLA-DQ2/8 epitopic presentation in CD, alluding to the potential protective role of those HLAs in fighting SARS-CoV-2 viruses.
7. HLA class II: Evolutionary protective mechanisms for CD survival. The wide range of COVID-19 manifestations, morbidity, and mortality seen across various ethnicities and geographical distribution was suggested to be host genetic dependent [131]. This genetic adaptative diversity may apply to CD. Interestingly, selective advantage mechanisms for polymorphic genes were speculated to contribute to the evolutionary survival of the CD populations [38,39,132] (Table 1). In fact, the human HLAs’ genetic heterogeneity is a known major anti-infectious mechanism to fight microbes, parasites, and even viruses, SARS-CoV-2 included. Although the variants of class II HLA loci were less frequently analyzed, they can impact COVID-19 outcomes. Most recently, HLA class II DRB1*01:01, DRB1*04:01, and DRB1*03:01 were reported to reduce disease duration and attenuated COVID-19 course [133,134,135]. Unfortunately, the HLA-DQ repertoire was not screened in those studies. Of note, the topic is still controversial and some studies denied the association between HLA polymorphisms and COVID-19 outcomes [136,137].

## 5. Celiac Disease and Long COVID-19 Syndromes

Most of the scientific studies were conducted on COVID-19 in CD, but the relationship between CD and the long COVID-19 syndrome is in its infancy. Nevertheless, typical symptoms like fatigue, poor appetite, abdominal pain, diarrhea, and nausea can overlap between the two entities [138,139]. Since most of the CD patients are undiagnosed, are a- or hypo-symptomatic, the question arises: Should the long COVID-19-affected patients be screened for CD? The jury is not yet out on that question. Intriguingly, nutritional deficiencies were proposed recently to impact COVID-19 and long COVID-19 outcomes [140]. One wonders if nutritional deficiencies during undiagnosed or gluten-free diet-treated CD patients [141,142] might subject them to long-term consequences of post-COVID-19 diseases. Most recently, Vojdani A et al. suggested that SARS-CoV-2 might activate latent Epstein–Barr virus and human herpesvirus 6, thus impacting the long COVID-19 phenotype [143]. Interestingly, both viruses were implicated as drivers of CD autoimmunity [143,144,145]. The question of whether HLA-DQ2 or 8 protects the CD patient population from long COVID-19 outcomes is still unresolved. Thus, despite the above-mentioned potential mechanistic pathways, the issue of HLA-DQ2/8–SARS-CoV-2 protective cross-talks is far from being deciphered.

## 6. Conclusions

**Figure 3:**
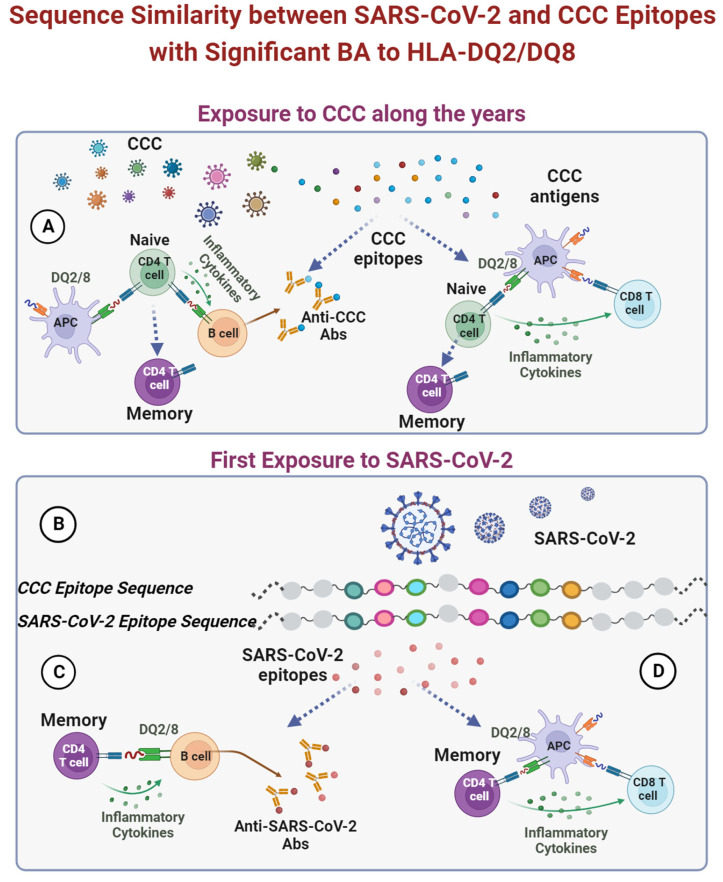
A schematic representation of 15-mer CCC epitopes with a minimum of 67% sequence identity to SARS-CoV-2 and strong binding to the celiac-associated HLA-DQ2/DQ8. (**A**) Exposure **to CCC**. Epitopes are presented, particularly on HLA-DQ2/8, to naïve CD4 T cells, leading to activation and proliferation, initiating an immune response. (**B**) Exposure to SARS-CoV-2. Some 15-mer epitopes have a minimum of 67% sequence identity to CCC and a significant BA- to CD-associated HLA-DQ2/8 (**C**,**D**). Those are presented to memory CD4 T cells, activating B cells, and CD8 T cells.

HLA class II genes are widely heterogenic, very polymorphic, and pivotal in presenting foreign antigens to T cells. Being important in fighting viral infection, the cross-talks between specific HLA II alleles and SARS-CoV-2 are important to understand the HLA genetic–COVID-19 outcome axes. Recent data are indicative of HLA-DQ2/8 protecting CD patients from SARS-CoV-2 infection or attenuating the COVID-19 course. We highlight the sequence similarity and the HLAs’ increased affinity as two novel mechanisms that might protect the CD patients from COVID-19 morbidity (Figure 3). Several potential mechanisms can be suggested to drive the phenomenon; however, the explanation is far from being elucidated. The present forwarded hypothesis that CD patients are protected from COVID-19 severity, morbidity, and associated acute and long-term complications should be further investigated.

## Figures and Tables

**Table 1:** Protective HLA variants in celiac disease.

Protective HLA Alleles	Country	References
HLA-DPB1*04:01	Germany, Finland, Sweden, and USA	[42]
HLA C14, DR11, DR15, DQ3	Turkey	[43]
Cw4 and DQ1	Spain	[44]
HLADQB1*06	Brazil	[45]
DRB1*13, DQA1*0102, DQB1*06	Tunisia	[46]
DQB1*0502/DQA1*0102	Sardinia	[47]
MICA-A5.1	Spain	[48]
MICA-A9	Basque country, Spain	[49,50]
DQA1*0101, DQA1*0201, DQB1*0301	Chile	[51]

**Table 2:** Intestinal features, events, and transmission of COVID-19.

Gastrointestinal Aspects	Enteric Features	References
Symptoms	Diarrhea, nausea, vomiting	[65,66,67]
ACE2 expression	All along the GIT	[68,69]
Inflammation and damage	Lymphocytic infiltration, edema, necrosis, degeneration, cellular shedding	[70,71]
Viral particles, nucleocapsid proteins	In the stomach, duodenum, and colonic cells	[70,71,72,73]
Stool shading	SARS-CoV-2 RNA in stool	[74,75,76,77]
Gut replication	SARS-CoV-2 multiplication in the gut	[76]
Feco–Oral transmission	Infectious virus is recovered from stool and urine samples.	[76,77,78,79]
Sewage, wastewater transmission	SARS-CoV-2 particles, RNA, and infectivity	[80,81]

**Table 3:** Sequence alignment between SARS-CoV-2 15-mer epitopes and CCC antigens and HLA-DQ2/8 binding affinity rank.

SARS-CoV-2 Parent Protein	CCC Protein ID	Epitope Sequence CCC vs. SARS-CoV-2 **	Identity	% Identity	Start	End	HLA-DQA10301-DQB10302 * Rank	HLA-DQA10501-DQB10201 * Rank
**NSP3 replicase polyprotein 1ab P0DTD1**	P0C6X5	SKDYISSNGPLKVG SDDYIATNGPLKVG	11/15	73.4%	1086	1100	4.28	
**NSP12 replicase polyprotein 1ab P0DTD1**	P0C6X2 P0C6X6 P0C6X1 P0C6X5	VVGVLTLDNQDLNGN IVGVLTLDNQDLNGN	14/15	93.3%	4593	4607		4.33
	P0C6X2	DFIQTAPGFGVAVAD DFIQTTPGSGVPVVD	11/15	73.3%	4613	4627	3.38	
		FIQTAPGFGVAVADS FIQTTPGSGVPVVDS	11/15	73.3%	4614	4628	1.15	4.18
		IQTAPGFGVAVADSY IQTTPGSGVPVVDSY	11/15	73.3%	4615	4629	0.76	2.75
		QTAPGFGVAVADSYY QTTPGSGVPVVDSYY	11/15	73.3%	4616	4630	0.63	2.24
	P0C6X2 P0C6X6	RQIFVDGVPFVVSIG RKIFVDGVPFVVSTG	13/15	86.7%	4723	4737		4.34
		KDLLLYAADPAMHVA KELLVYAADPAMHAA	12/15	80.0%	4761	4775		4.3
	P0C6X6	TSGVKFQTVKPGNFN TNNVAFQTVKPGNFN	12/15	80.0%	4794	4808		3.03
		SGVKFQTVKPGNFNQ NNVAFQTVKPGNFNK	11/15	73.3%	4795	4809		1.6
	P0C6X2 P0C6X6	VKFQTVKPGNFNQD VAFQTVKPGNFNKD	12/15	80.0%	4796	4810		0.7
		VKFQTVKPGNFNQDF VAFQTVKPGNFNKDF	13/15	86.7%	4797	4811		2.98
		FFFTQDGNAAITDYN FFFAQDGNAAISDYD	12/15	80.0%	4832	4846	2.9	
		FFTQDGNAAITDYNY FFAQDGNAAISDYDY	12/15	80.0%	4833	4847	1.98	
		FTQDGNAAITDYNYY FAQDGNAAISDYDYY	12/15	80.0%	4834	4848	2.12	
	P0C6X5 P0C6X1 P0C6X2 P0C6X6	SSGDATTAFANSVFN SSGDATTAYANSVFN	14/15	93.3%	5073	5087	4.91	2.73
	P0C6X2	DYVYLPYPDPS DYVYLPYPDPS	11/15	73.3%	5213	5227	4.02	
	P0C6X5 P0C6X1 P0C6X2 P0C6X6	LLIERFVSLAIDAYP LMIERFVSLAIDAYP	14/15	93.3%	5246	5260	3.36	1.42
		LIERFVSLAIDAYPL MIERFVSLAIDAYPL	14/15	93.3%	5247	5261	4.39	1.75
		IERFVSLAIDAYPL IERFVSLAIDAYPL	14/15	93.3%	5248	5262		1.53
		ERYVSLAIDAYPLSK ERFVSLAIDAYPLTK	13/15	86.7%	5249	5263		3.4
**NSP13 replicase polyprotein 1ab P0DTD1**	P0C6X5 P0C6X1	PEVNADIVVVDEVSM PETTADIVVFDEISM	11/15	73.3%	5688	5702	1.08	
	P0C6X2 P0C6X6	RAKHYVYIGDPAQLP RAKHYVYIGDPAQLP	15/15	100.0%	5716	5730		3.24
	P0C6X5 P0C6X1 P0C6X2 P0C6X6	AKHYVYIGDPAQLPA AKHYVYIGDPAQLPA	15/15	100.0%	5717	5731		2.45
		KHYVYIGDPAQLPAP KHYVYIGDPAQLPAP	15/15	100.0%	5718	5732		3.52
		CPKEIVDTVSALVYE CPAEIVDTVSALVYD	13/15	86.7%	5768	5782		3.27
		PKEIVDTVSALVYEN PAEIVDTVSALVYDN	13/15	86.7%	5769	5783	2.67	1.3
		EIVDTVSALVYENK EIVDTVSALVYDNK	13/15	86.7%	5770	5784	1.09	0.65
		EIVDTVSALVYENKL EIVDTVSALVYDNKL	14/15	93.3%	5771	5785	1.61	2.77
	P0C6X6 P0C6X2	IVETVSALVYDNKLK IVDTVSALVYDNKLK	14/15	93.3%	5772	5786	4.2	
	P0C6X5 P0C6X1 P0C6X2 P0C6X6	EYDYVIYSQTAETAH EYDYVIFTQTTETAH	12/15	80.0%	5864	5878		3.76
	P0C6X1 P0C6X2 P0C6X6	YDYVIYSQTAETAHS YDYVIFTQTTETAHS	12/15	80.0%	5865	5879		3.07
	P0C6X1 P0C6X5 P0C6X2 P0C6X6	TAETAHSVNVNRFNV TTETAHSCNVNRFNV	13/15	86.7%	5873	5887		3.43
		ETAHSVNVNRFNVA ETAHSCNVNRFNVA	13/15	86.7%	5874	5888		2.98
**Spike glycoprotein P0DTC2**	Q5MQD0	FGAISSSLQEILSR FGAISSVLNDILSR	11/15	73.4%	969	983		4.86
	P36334 Q5MQD0	DALEAQVQIDRLING DKVEAEVQIDRLITG	11/15	73.3%	985	999	4.45	4.48
		DALEAQVQIDRLING DPPEAEVQIDRLITG	11/15	73.3%	985	999	2.93	3.24

* The two columns on the right-hand-side display the binding rank. Those ranked in the top 1% were classified as potential SB and those ranked in the top 5% were classified as WB. ** Identical AAs are marked in red.

## Data Availability

The data and software that support the findings of this study are openly available in: The Immune Epitope Database (IEDB) at www.iedb.org [99,146] on 18 August 2023; UniProt Knowledgebase www.uniprot.org [98] on 17 August 2023; Pairwise Local Alignment tool, EMBOSS Matcher, at www.emboss.sourceforge.net [102,103]; A python script can be found at https://raw.githubusercontent.com/ebi-wp/webservice-clients/master/python/emboss_matcher.py (version 2.0); Binding Affinity Prediction tool from DTU Health Tech, the NetMHCIIpan-4.2 method, at https://services.healthtech.dtu.dk/services/NetMHCIIpan-4.2/ [106] (27 August 2023).

## Abbreviations

GIT—gastrointestinal tract, CD—celiac disease, IEDB—Immune Epitope Database and Analysis Resource, CCC—common cold coronaviruses.
